# Phenotypic response to different predator strategies can be mediated by temperature

**DOI:** 10.1002/ece3.10474

**Published:** 2023-08-31

**Authors:** Francesco Cerini, Duncan O'Brien, Ellie Wolfe, Marc Besson, Christopher F. Clements

**Affiliations:** ^1^ Dipartimento Scienze Ecologiche e Biologiche Università della Tuscia Viterbo Italy; ^2^ School of Biological Sciences University of Bristol Bristol UK; ^3^ Sorbonne Université CNRS UMR Biologie des Organismes Marins, BIOM Banyuls‐sur‐Mer France

**Keywords:** antipredatory responses, gantry, microcosms, *Paramecium caudatum*, predation strategies, protists

## Abstract

Temperature change affects biological systems in multifaceted ways, including the alteration of species interaction strengths, with implications for the stability of populations and communities. Temperature‐dependent changes to antipredatory responses are an emerging mechanism of destabilization and thus there is a need to understand how prey species respond to predation pressures in the face of changing temperatures. Here, using ciliate protozoans, we assess whether temperature can alter the strength of phenotypic antipredator responses in a prey species and whether this relationship depends on the predator's hunting behavior. We exposed populations of the ciliate *Paramecium caudatum* to either (i) a sit‐and‐wait generalist predator (*Homalozoon vermiculare*) or (ii) a specialized active swimmer predator (*Didinium nasutum*) across two different temperature regimes (15 and 25°C) to quantify the temperature dependence of antipredator responses over a 24‐h period. We utilized a novel high‐throughput automated robotic monitoring system to track changes in the behavior (swimming speed) and morphology (cell size) of *P. caudatum* at frequencies and resolutions previously unachievable by manual sampling. The change in swimming speed through the 24 h differed between the two temperatures but was not altered by the presence of the predators. In contrast, *P. caudatum* showed a substantial temperature‐dependent morphological response to the presence of *D. nasutum* (but not *H. vermiculare*), changing cell shape toward a more elongated morph at 15°C (but not at 25°C). Our findings suggest that temperature can have strong effects on prey morphological responses to predator presence, but that this response is potentially dependent on the predator's feeding strategy. This suggests that greater consideration of synergistic antipredator behavioral and physiological responses is required in species and communities subject to environmental changes.

## Graphical abstract

*Paramecium caudatum* shows a substantial temperature‐dependent morphological response to the presence of *Didinium nasutum* (but not Homalozoon vermiculare), changing cell shape toward a more elongated morph at 15°C (but not at 25°C). This suggests that temperature can have strong effects on prey morphological responses to predator presence, but that this response is potentially dependent on the predator's feeding strategy.

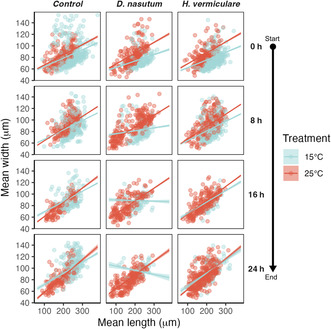

## 1 INTRODUCTION

Individuals' capacity to withstand challenges in their environment is directly related to their ability to alter their phenotype (i.e., phenotypic plasticity; Cerini et al., 2023; Chevin et al., 2013). In organisms experiencing predation (i.e., prey species), evolution shapes mechanisms of predator detection and avoidance, resulting in behavioral and morphological changes that are observed across all biological scales, from protozoa to metazoa (Bourdeau & Johansson, 2012; Fyda et al., 2005; Hammill et al., 2009; Luhring et al., 2019). In addition to the obvious advantage for prey survival, predator‐induced responses reduce the maximum number of prey that predators can eat (Altwegg et al., 2006), thus stabilizing ecosystem dynamics and services (Madritch & Hunter, 2002).

Antipredatory responses or adaptations can be induced and/or altered by multiple aspects of a prey's environment. Most obviously, changes can occur in response to a predator's presence via visual, acoustic, physical, or chemical cues (Fischer & Frommen, 2019). Upon detection, these cues drive immediate (e.g., behavioral) responses in the prey or the expression of genes regulating morphological defensive features (i.e., induced responses; Tollrian & Harvell, 1999; Weiss, 2019) to decrease predation risk. Global climate changes in temperature and atmospheric CO_2_ can, however, influence cue detection, especially in aquatic environments where physical and chemical cues are dominant (Draper & Weissburg, 2019). Chemical cues are molecules released by the predator and “perceived” by prey, either via ingestion and olfaction in multicellular organisms or cell surface receptors in unicellular organisms (Kusch, 1993, Wicklow, 1997). Temperature alters the diffusive and biochemical behavior of these chemical cues, which will in turn influence the antipredator changes expressed by a stressed prey population. For example, higher temperatures allow chemical cues to diffuse faster but may change absorbance behavior or degrade (Chivers et al., 2013) in potentially unexpected ways.

Similarly, climate change can directly or indirectly alter both prey morphological and behavioral traits involved in antipredatory functions. For example, increased water temperature and acidification can degrade bivalve shell integrity (Lemasson & Knights, 2021) or impair avoidance behaviors and predation susceptibility in prey fish (Besson et al., 2020; Paul et al., 2021). Given the rapid temperature increases faced by natural populations (Field & Barros, 2014), there is a need to understand whether temperature increases compromise, aid, or maintain antipredatory morphological and behavioral responses and how any impacts shape the dynamics and resilience of ecological communities.

Experimental populations of model organisms are valuable tools to tackle these general questions. Due to their high flexibility and small scale, one can perform accurate manipulations of temperature, population density, and predator presence while enabling a high degree of replication at a low cost of time and resources (Altermatt et al., 2015). Ciliate protozoa have facilitated a rich body of literature on the effects of predator presence on both prey morphology and behavior. Responses are driven by direct physical contact or, mostly, by chemical cues (i.e., proteins) released by the predator that meet receptors on the ciliate cells, triggering the reactions (Wicklow, 1997). Behavioral antipredatory responses in prey ciliates are primarily associated with their swimming speed or number of directional changes (Kusch, 1993), with patterns depending on their predators' feeding ecology. For example, chemical cues of a swimming flatworm predator induce a decrease in the velocity of *Euplotes* and *Paramecium*, potentially lowering the rates of predator encounters (Hammill, Kratina, et al., 2010; Hammill, Petchey, & Anholt, 2010), while the presence of a slow crawler predator (*Amoeba*) triggers active avoidance behaviors with increased turning rates in predator's vicinity (Kusch, 1993). Such behavioral changes can occur with, or be followed by, shifts in morphology (Hammill, Kratina, et al., 2010). Exemplary ciliate morphological changes include the development of spine‐shaped cytoplasmatic projections (Altwegg et al., 2006; Kuhlmann & Heckmann, 1994) and increases in the length and/or width of individuals (Fyda et al., 2005; Hammill, Petchey, & Anholt, 2010). Size increase can be a prey morphological response to the presence of gape‐limited predators (e.g., *Stenostomum*, Altwegg et al., 2006), whereby the prey can become too large for the predators to ingest or more difficult to interact with (i.e., extended handling time of prey; Hammill, Petchey, & Anholt, 2010). Prey morphological responses can also vary with predator strategy. For example, as a response to a nongape‐limited and actively swimming predator (e.g., Copepoda), the ciliate *Paramecium caudatum* develops a narrower shape that may maximize maneuverability and the escape probability from predators' attack (Uiterwaal et al., 2020).

This range of prey behavioral and morphological responses is less predictable when predator presence is combined with temperature change. Environmental temperature has strong effects on basal metabolism (Weber de Melo et al., 2020), but its effects on prey behavior and morphological dependencies are unclear. Theory predicts that increased temperatures lead to increased swimming activities in both prey and predators (Dell et al., 2014). This has been evidenced in two *Paramecium* species, which increased their swimming speed when exposed to higher temperatures (in both predator‐exposed and no‐predator scenarios; Robertson & Hammill, 2021). In a different experiment at a stable temperature, one of the two *Paramecium* species studied by Robertson and Hammill (2021) responded to chemical cues from the same predator flatworm by decreasing its speed (Hammill, Petchey, & Anholt, 2010). Higher temperatures can therefore potentially mask or reverse antipredatory responses, but this has not been specifically clarified with consistency using model species. In terms of morphological responses, higher temperatures are generally associated with smaller sizes in protists (Atkinson, 1994; Atkinson et al., 2003; Weber de Melo et al., 2020), although other work showed that ciliate body length was not influenced by temperature, regardless of acclimation (Beveridge et al., 2010). This indicates that a single measure such as cell length may not be sufficient to relate temperature to morphological changes and that multiple morphological measures may be more appropriate. Indeed, a measure of the length: width ratio revealed a trade‐off in *P. caudatum* morphological responses to temperature versus predation, with individuals becoming rounder (decreased length:width ratio) in response to variable temperatures (probably as an adaptive response for optimizing gas and resource exchanges), while becoming more elongated (increased length:width ratio) in response to generalist predator exposure (potentially as an adaptative response to optimize predation avoidance, Uiterwaal et al., 2020). Nevertheless, such literature remains hardly generalizable, and little is known regarding how temperature stress and predator feeding ecology (e.g., sit‐and‐wait vs. chasers, generalists vs. specialists) interact to shape prey behavioral and morphological antipredatory adaptations.

We tackled this gap by analyzing and comparing swimming speeds and individual cell size of the ciliate *P. caudatum* at two different temperatures and under three predator conditions by means of a novel high‐throughput automated monitoring system (Besson et al., 2022) that allows us to monitor the behaviors and morphologies of experimental ciliates in an automated and reproducible fashion without disturbing the communities.

## 2 METHODS

### 2.1 Laboratory settings and protocol

We exposed populations of *P. caudatum* to two predators with different hunting strategies (*Didinium nasutum*—highly mobile— and *Homalozoon vermiculare—*sit and wait) at two temperatures (15°C and 25°C) and tracked changes in *P. caudatum* behavior (swimming speed) and morphology (body dimensions). All species were maintained as laboratory stocks at the University of Bristol and were originally obtained from Sciento (Manchester, UK).

Prey were exposed to predators in rectangular experimental microcosms (5.5 × 3.5 × 2 cm), custom designed using FreeCAD 3D‐design software (https://www.freecad.org/), and 3D‐printed in clear PLA filament (Lulzbot TAZ 6). The base of each microcosm was painted with black acrylic paint and coated in clear epoxy resin to smooth the patch surface and prevent potential paint leaching. Each microcosm had a capacity of 5 mL of water‐based media. The media consisted of crushed protozoa pellets (Blades Biological LTD) dissolved in Chalkley's solution at a concentration of 0.3 g L^−1^, then autoclaved (Clements et al., 2013). The media was then filtered through Whatman n°1 filter paper to improve media clarity and autoclaved again. This medium was inoculated with two species of bacteria—*Bacillus subtilis* and *Pseudomonas fluorescens*—24 h prior to experiment set‐up. We acclimated all stocks to the experiment temperatures for 1 week prior to the experiment and starved predators for 24 h to standardize satiation levels.

Experiments were initiated by first adding 4.8 mL bacterized media to each microcosm, followed by 25 *P. caudatum* individuals in 150 μL of media. The timing of *P. caudatum* addition was carefully timed by staggering addition in 90‐s intervals so that each microcosm had *P. caudatum* added exactly 5 min before the first sampling event (see below), which occurred before the predator addition, to create a baseline of morphological and behavioral measurements. This ensures any changes in *P. caudatum* behavior or morphology recorded immediately after predator addition occurred because of predator presence rather than the disturbance of being added to the microcosm. One hour after the initial *P. caudatum* addition, 5 min prior to the second sampling event (see below), we added the predators—five individuals of either *D. nasutum* or *H. vermiculare* in 50 μL media or 50 μL bacterized media (prey alone treatment)—to each patch. This process was also conducted in a staggered fashion to ensure the predators or controls were added 5 min prior to the sampling of that microcosm. The addition of bacterized media to the prey alone treatment controlled for the disturbance caused by adding more volume to the microcosm and ensured all microcosms contained the same final volume of 5 mL media. Each treatment was replicated ten times at each of the two temperatures: 15°C and 25°C. To achieve this, microcosms were placed in a climate‐controlled room at 80% humidity to lower evaporation rates. A 10°C temperature difference is sufficient to cause changes in the growth rate and carrying capacity of *P. caudatum* (Wolfe et al., 2022), but does not induce a strong change in the water viscosity. This is important as viscosity constrains speed at low temperatures (Beveridge et al., 2010), thus potentially masking antipredatory shifts in behavior. During our experiment, the low density of our *D. nasutum* stock allowed us to perform only six replicates instead of ten. Additionally, in two replicates of the “prey alone” treatment at 25°C, all the individuals died rapidly after setting up for unknown reasons, so those replicates were excluded.

**FIGURE 1:**
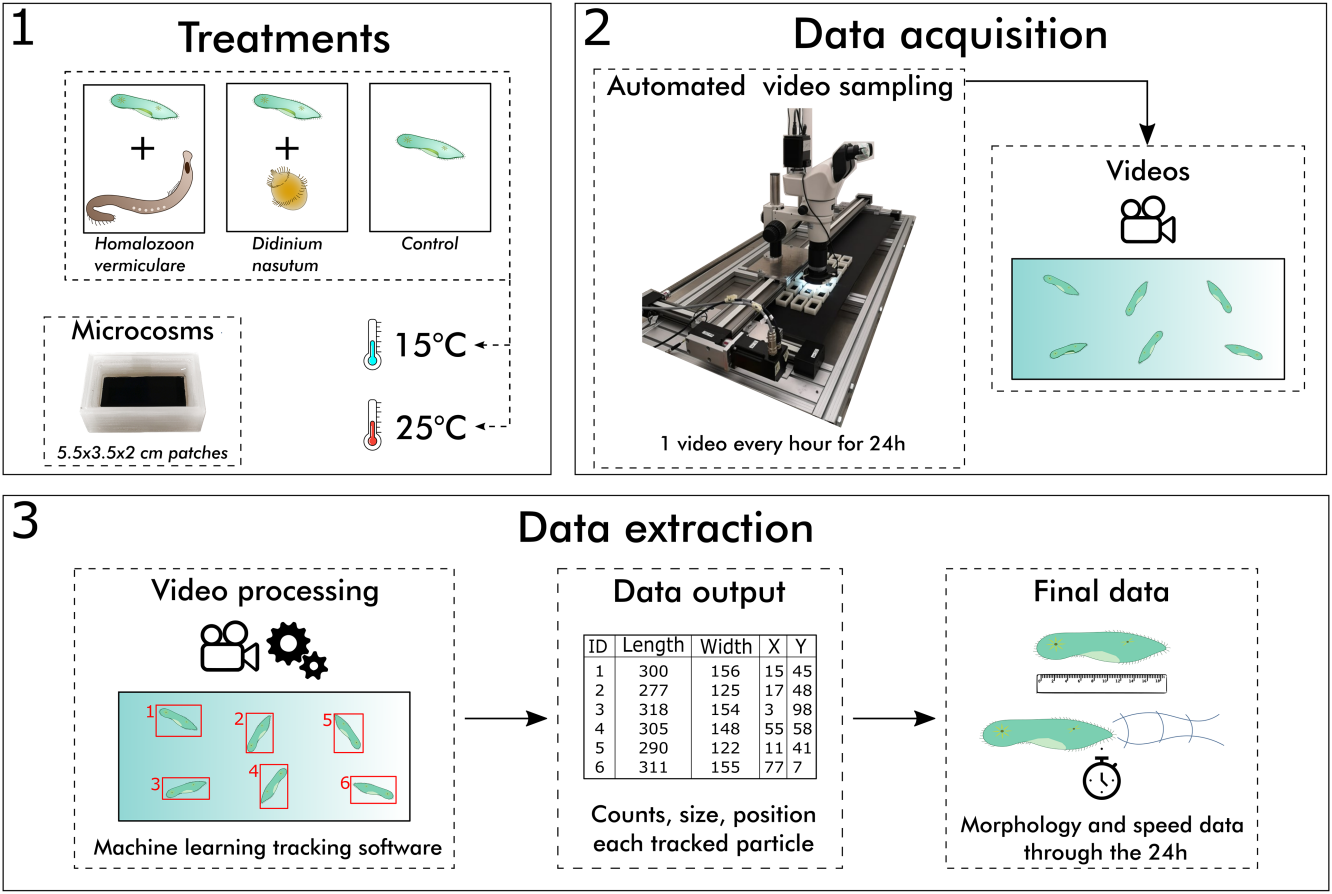
Experiment conceptual pipeline. (1) The three treatments with prey exposed to *Homalozoon vermiculare*, *Didinium nasutum*, or no predator in the microcosms, consisting of rectangular PLA patches. The three treatments were repeated at 15°C and at 25°C. (2) Video recording was automated by means of a robotic gantry moving a microscope connected to a camera, with a frequency of 1 video every hour for 24 h for each replicate. (3) The videos were processed with tracking software that gives back the position in bidimensional space and the size of each moving particle, using machine learning to recognize the different species. From such output, we extracted average speeds and morphological data for each ciliate tracked in each video through the 24 h.

After their addition to the microcosms, *P. caudatum* was monitored once every hour for 24 h. This monitoring was carried out using an automated tracking system consisting of a camera (GXCAM HighChrome‐HR4 HI RES) connected to a stereomicroscope (Nikon SMZ1270) attached to a robotic gantry (igus drylin Gantry) programmed to record 12‐s videos of each microcosm every hour for the duration of the experiment (24 h, Figure 1). Once all videos had been recorded and experiments completed, we applied ComTrack, an open‐source machine learning‐based software designed to extract individual morphological and behavioral information, as well as species abundances and spatial distributions, from videos containing multiple species (Besson et al., 2021). These data allowed us to extract information on the speed, body length, and width of every tracked individual of *P. caudatum* and the two predator species at every time point through time (Figure 1).

### 2.2 Statistical analyses

We first visualized the abundance counts of *P. caudatum* through time to understand if the ciliates in the predator treatments were quickly wiped out (Figure S1) and confirmed that the numbers of tracked individuals did not differ strongly from the control treatments. Then, to explore the effect of predator presence on *P. caudatum* behavior, we calculated the average speed of each individual per 12‐s video. Thus, each tracked ciliate had a mean value of speed, length, and width for each time point (24 time points) across all the treatments and replicates. We then compared the trend of the mean speed through the 24 h between control and the predator treatments in the two temperature settings by means of a multilevel generalized additive Bayesian model.

The resulting model structure was:

$$y_{\mathrm{ij}}\sim \text{weibull}\left(\mu_{\mathrm{ij}},\Gamma \left(1+1/\ln(\text{shape}\right)\right))$$

$$\begin{array}{cc} \mu_{\mathrm{ij}}=\; & \beta_{0j}+\beta_{1}{\text{Temperature}}_{i}+\beta_{2}{\text{Predator}}_{i}\\ & +\beta_{3}{\text{Temperature}}_{i}:{\text{Predator}}_{i}+s_{1}\left({\text{Time}}_{i}\right)\\ & +s_{2}\left({\text{Time}}_{i},{\text{Temperature}}_{i}\right)+s_{3}\left({\text{Time}}_{i},{\text{Predator}}_{i}\right)\\ & +s_{4}\left({\text{Time}}_{i},{\text{Predator}}_{i}:{\text{Temperature}}_{i}\right)+\varepsilon_{\mathrm{ij}} \end{array}$$

$$\beta_{0j}\sim \text{Normal}\left(\beta_{0},\sigma_{\beta_{0}}\right)$$

$$s\left(x\right)=\sum_{k=1\ldots 8}^{K}\beta_{k}b_{k}\left(x\right)$$

where *y*_*ij*_ represents the mean speed in the *i*th *P. caudatum* observation of the *j*th replicate. *Μ*_*ij*_ is therefore the expected value for each observation, with *β*_1–3_ linear coefficients and *β*_0_ random intercepts per replicate *j*. *Γ* represents the gamma function, while *s*_1–4_(*x*) are smooth thin‐plate splines with a basis function *b*_*k*_ and coefficients *β*_*k*_. The maximum complexity of these splines is *K*, but it is penalized to minimize *k*. *ε*_*ij*_ represents the remaining error.

We then set the weakly informative priors:

$$\beta_{0}\sim \text{exponential}\left(1\right)$$

$$\beta \sim \text{Normal}\left(0,1\right)$$

$$\sigma \sim \text{exponential}\left(1\right)$$

$$\sigma_{\beta_{0}}\sim \text{exponential}\left(1\right)$$

$$\text{shape}\sim \text{gamma}\left(0.01,0.01\right)$$

We also explored the effect of predator presence on the relationship between the mean width and length of the *P. caudatum* individuals for each temperature and predator treatment. We analyzed how this relationship changed throughout the experiment's 24 hours using a Bayesian linear mixed effect model with the following structure:

$$y_{\mathrm{ij}}\sim \text{Normal}\left(\mu_{\mathrm{ij}},\sigma \right)$$

$$\begin{array}{cc} \mu_{\mathrm{ij}}=\; & \beta_{0j}+\beta_{1}{\text{Length}}_{i}+\beta_{2}{\text{Temperature}}_{i}+\beta_{3}{\text{Predator}}_{i}+\beta_{4}{\text{Time}}_{i}\\ & +\beta_{5}{\text{Length}}_{i}:{\text{Predator}}_{i}+\beta_{6}{\text{Length}}_{i}:{\text{Temperature}}_{i}\\ & +\beta_{7}{\text{Length}}_{i}:{\text{Time}}_{i}+\beta_{8}{\text{Temperature}}_{i}:{\text{Predator}}_{i}\\ & +\beta_{9}{\text{Temperature}}_{i}:{\text{Time}}_{i}+\beta_{10}{\text{Predator}}_{i}:{\text{Time}}_{i}\\ & +\beta_{11}{\text{Length}}_{i}:{\text{Temperature}}_{i}:{\text{Predator}}_{i}+\beta_{12}{\text{Length}}_{i}:{\text{Temperature}}_{i}:{\text{Time}}_{i}\\ & +\beta_{13}{\text{Length}}_{i}:{\text{Predator}}_{i}:{\text{Time}}_{i}+\beta_{14}{\text{Temperature}}_{i}:{\text{Predator}}_{i}:{\text{Time}}_{i}\\ & +\beta_{15}{\text{Length}}_{i}:{\text{Temperature}}_{i}:{\text{Predator}}_{i}:{\text{Time}}_{i}+\varepsilon_{\mathrm{ij}} \end{array}$$

$$\beta_{0j}\sim \text{Normal}\left(\beta_{0},\sigma_{\beta_{0}}\right)$$

where *y*_*ij*_ represents the mean width (μm) in the *i*th *P. caudatum* observation of the *j*th replicate. *μ*_*ij*_ is therefore the expected value for each observation, with *β*_1–15_ linear coefficients, *β*_0_ random intercepts per replicate *j* and *ε*_*ij*_ the remaining error.

We then set the weakly informative priors:

$$\beta_{0}\sim \text{Normal}\left(50,10\right)$$

$$\beta \sim \text{Normal}\left(0,1\right)$$

$$\sigma \sim \text{exponential}\left(1\right)$$

$$\sigma_{\beta_{0}}\sim \text{exponential}\left(1\right)$$

All models were run for 10,000 iterations, with a warmup of 2000 iterations. We assessed convergence visually by examining trace plots, posterior predicting checks, and Rhat values (the ratio of the effective sample size to the overall number of iterations, with values close to one indicating convergence). To have an indication of the difference in predator activity levels (i.e., movement speed), we compared the average swimming speeds in the two temperature treatments via a Wilcoxon test. All analyses were performed in R (R version 4.1.2, https://cran.r‐project.org/) with the use the of *brms* package (Bürkner, 2021).

## 3 RESULTS

Our dataset consisted of 499,013 single‐frame‐based measurements of the position and morphology of *P. caudatum* individuals. Once these observations were averaged per video (i.e., per time point), we had a final pool of 25,059 mean values of speed and morphology across the treatments and through the 24 h.

The change in numbers of tracked *P. caudatum* through the hours of the experiments was generally similar across the predators and the control treatments and between the two temperatures, with some variation across replicates of the same treatments (Appendix S1: Figure S1). At 15°C, the mean number of surviving preys by the end of both the *D. nasutum* and *H. vermiculare* treatments was slightly lower than the control, but without a strong difference in the average trend through time (Appendix S1: Figure S1). Nevertheless, predation occurred, and there were fewer surviving individuals in the predator's treatments (*D. nasutum* 6.74; *H. vermiculare* 7.31; control 9.72). Such an offset was not observed between the 25°C control and the *D. nasutum* treatment, while the *H. vermiculare* treatment on average maintained slightly higher numbers than the control. The biggest difference was found in the *H. vermiculare* treatment across the two temperatures, whereby the replicates at 25°C maintained on average a higher number of surviving preys through time (Appendix S1: Figure S1). For the control and *D. nasutum* treatments, we did not observe evident inter‐temperature setting differences.

**FIGURE 2:**
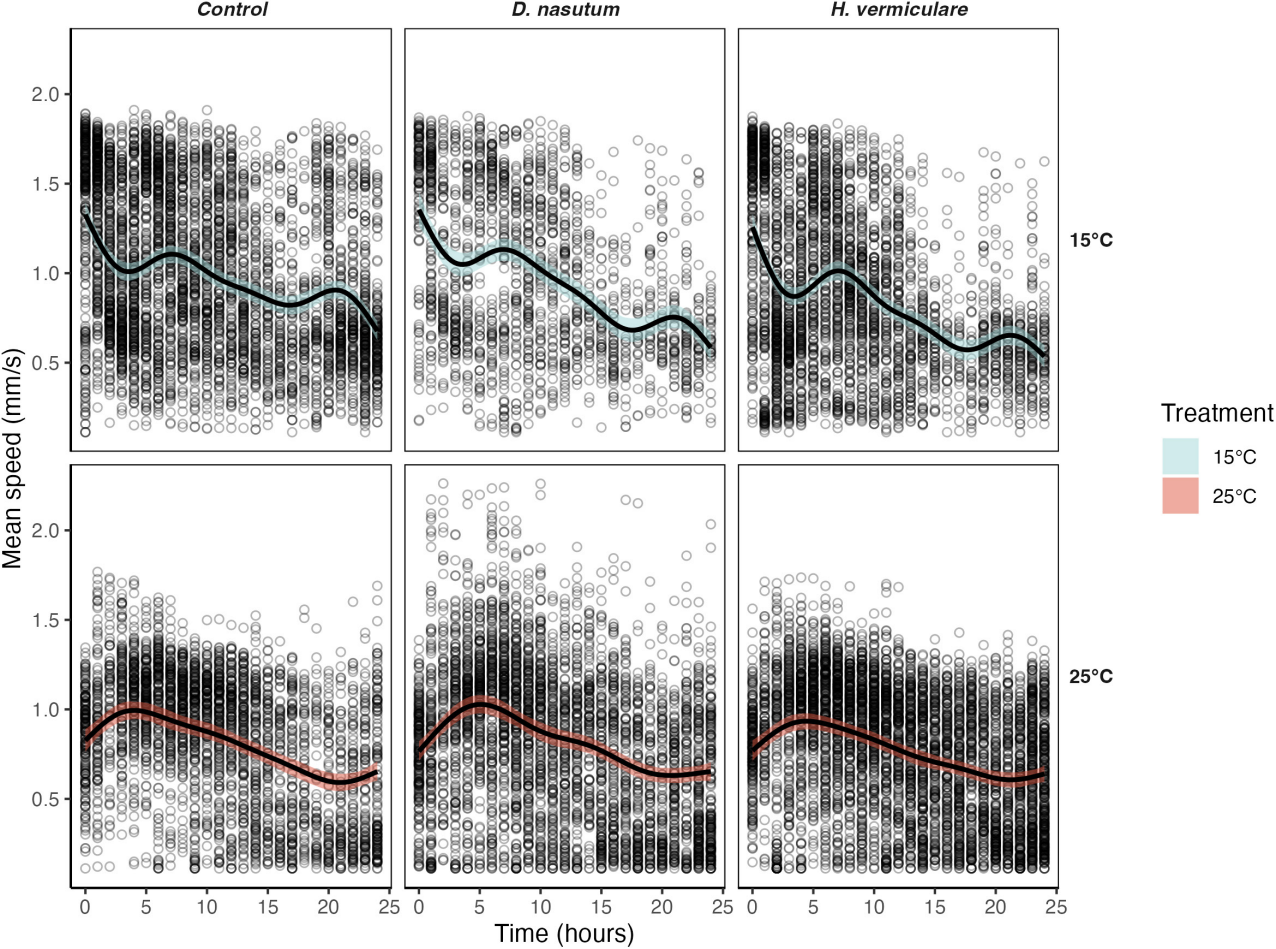
Estimated marginal effect smooths of average *Paramecium caudatum* speeds through time and across temperature and predator treatments. Points represent the raw data; black lines represent the estimated median; smooth and shaded region (blue and red) represent the 95% credible interval. The control treatment consists of *P. caudatum* alone, whereas the two other predator treatments introduce a set number of each predator species.

For model diagnostics of the following results, please refer to Appendix S1: Figures S2–S5. The overall swimming speed of *P. caudatum* differed slightly between the two temperatures (Figure 2, Appendix S1: Table S1), but predator presence had no effect on this trait relative to the control treatment, neither at 15°C nor at 25°C. Similarly, the degree of smoothing (i.e., the level of variability through time) differed between temperature treatments but was consistent between predator treatments of the same temperature treatment (Figure 2, Appendix S1: Table S1). At both temperatures, regardless of the predators, *P. caudatum* showed a slight decline in speed over the 24 h compared to the initial average speed values. However, there was a more pronounced difference during the first 5 h, for which we observed a steep speed decline for the 15°C treatment and a slight increase in the 25°C treatment, compared to the swimming speed at time 0 (Figure 2).

*Didinium nasutum* individuals showed higher average swimming speeds in the 25°C treatment (*W* = 10,044, *p* < .01, Appendix S1: Figure S5). *H. vermiculare* did not show any difference in the average speeds between the two temperatures (*W* = 5526, *p* = .316, Appendix S1: Figure S5).

**FIGURE 3:**
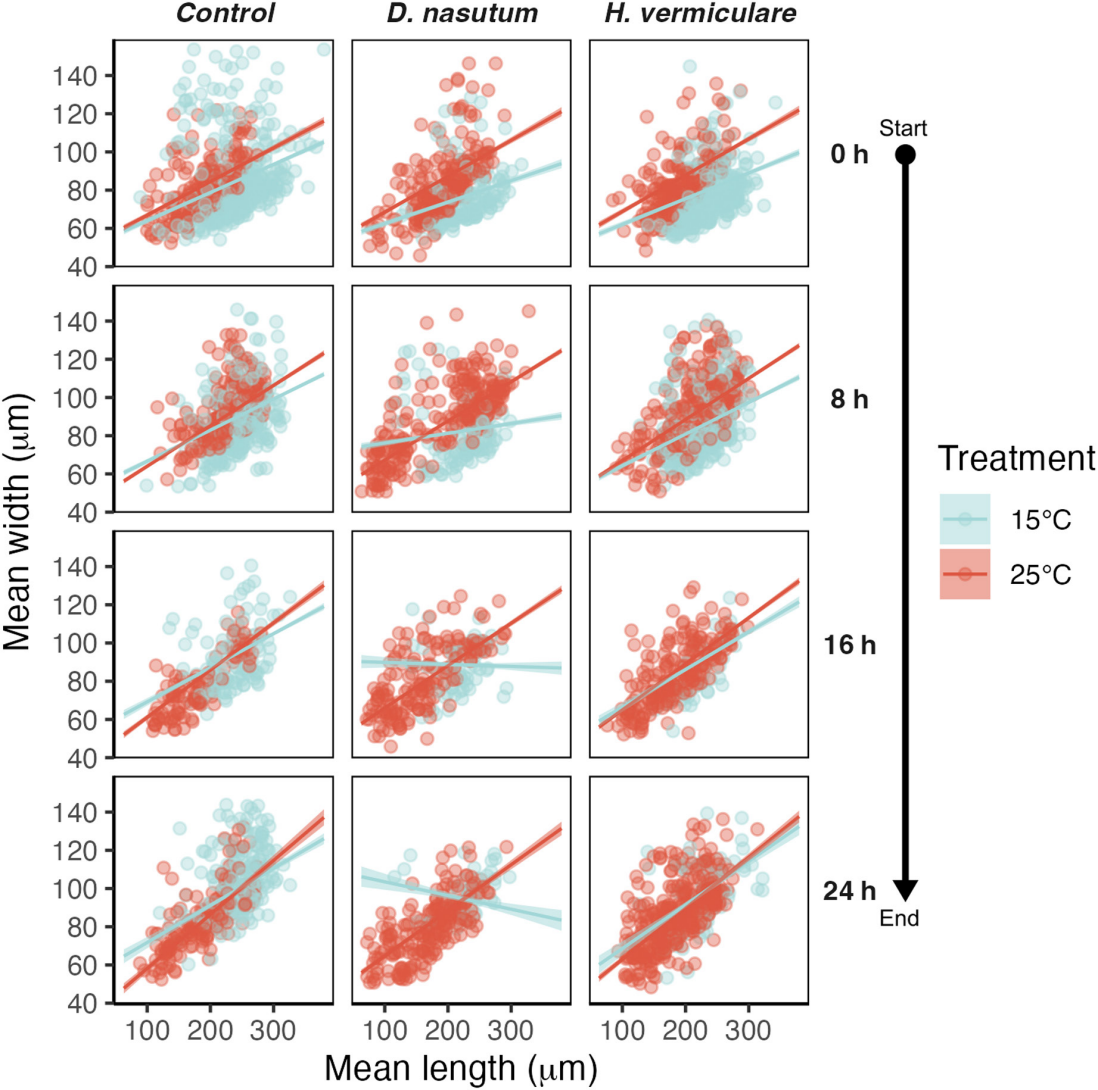
Estimated marginal effects of *Paramecium caudatum* morphology through time and across temperature and predator treatments. Four time points (the four rows of panels) are displayed for simplicity: from the start to the end of the experiment with 8 h gaps (i.e., time points 0, 8, 16, 24). Points represent the raw data; solid lines represent the estimated median; smooth and shaded region represent the 95% credible interval. The Control treatment consists of *P. caudatum* alone, whereas the two other predator treatments introduce a set number of each predator species.

The regression between *P. caudatum* width and length over time showed a significant interaction of the *D. nasutum* predator treatment with temperature. Specifically, at 15°C, the *D. nasutum* treatment resulted in a change of slope and intercept through time, with individuals changing toward a more elongated shape (higher length values at parity of width values, Figure 3). This elongation response was first observed after 8 h of *D. nasutum* exposure (Figure 3). In contrast, the regression in the 15°C treatment only showed minor changes in the control and the *H. vermiculare* treatments (i.e., no significant interaction; Figure 3, Appendix S1: Table S2). Similarly, the slope and intercept of the regression did not change through time in the 25°C treatments, regardless of the predator's species presence or absence (Figure 3, Appendix S1: Table S2).

## 4 DISCUSSION

Understanding how climate change can impact ecosystem dynamics requires an investigation of how basic evolutionary adaptations might change at different temperatures. Prey phenotypic (e.g., behavioral and morphological) responses to predator presence are among the plasticity mechanisms shaped by shifts in temperature, with conflicting evidence across taxa; some reactions are amplified (e.g., predator inspection in fish [Weetman et al., 1998]), while others are unaffected (e.g., antipredatory display in snakes [Brodie III & Russell, 1999]). With the aim of gaining insights on this complex topic, we tackled the question using an experimental and highly replicable approach. By means of a novel system that allowed us to measure behavior and morphology in a noninvasive fashion and at high resolution (Besson et al., 2021), we monitored populations of ciliate protozoa in microcosms exposed to predators with different feeding strategies across two temperature treatments. Our results demonstrate that, in our study system, elevated temperatures can affect prey morphological responses to predator presence, potentially inducing a change in the antipredatory value of such responses. However, the occurrence of morphological responses depends on the predator's feeding ecology.

Relationships between temperature, predator ecology, and prey response can obscure our ability to accurately predict prey dynamics in a changing environment if potential interactive effects are not identified. Here, we show that only certain prey responses are the result of interactions between multiple stressors (temperature and predator ecology), whereas in the noninteractive scenarios, prey responses are dictated by environmental conditions. This matches the traditional understanding that the environment enforces predator–prey interaction strengths regardless of taxa (e.g., mollusks and crustaceans [Smee & Weissburg, 2006]; fish and crustaceans [Lunt & Smee, 2015]; mammals [Tablado et al., 2014]) and drives consistent responses in both trophic levels. However, we also present an example of a temperature‐inhibited morphological response that only occurred in response to a specific predator ecology. Some examples exist of differential predator foraging ability across environmental conditions (Powers & Kittinger, 2002), but here we suggest that prey response is also important and arguably more vulnerable to environmental stress. This is particularly relevant to morphological or physiological antipredatory responses, which are constrained within biomechanical and biochemical norms.

Despite clear observations in previous work on the same *P. caudatum* model showing a speed shift after exposure to predators (Hammill, Petchey, & Anholt, 2010), we did not observe any changes in swimming speeds between the predator treatments. In fact, although the average speeds changed over time across the two temperatures, we did not observe any differences between the control and the predator treatments. Previous tests where a clear decrease in swimming speed was observed in the presence of predator chemical cues were performed in much smaller habitats compared to our system (e.g., less than 1 mL well vs. 5 mL microcosm in our study), with a much higher density of predator cues than in natural systems (Hammill, Petchey, & Anholt, 2010). One possibility is that our microcosm volume might be too large for the presence of indirect predator cues (i.e., not coming from direct interactions) to trigger swimming speed changes in prey. Conversely, the responses previously observed under exposure to a more complex predator's chemical cues may not be generalizable to single‐cell predators like *D. nasutum* and *H. vermiculare*, even though other ciliate prey species expressed behavioral changes induced by ciliate predator cues (Kusch, 1993). A slower swimming pattern may be advantageous for the prey in the case of an active predator with a gape‐limited mouth like the flatworms (Hammill, Kratina, et al., 2010; Hammill, Petchey, & Anholt, 2010), as the predator's extended handling time can allow prey escape (Nuttycombe & Waters, 1935). However, in the presence of *D. nasutum* and *H. vermiculare*, any response that decreases the likelihood of meeting the predator is counterbalanced by the risk of being more vulnerable to a lethal toxicyst strike. We suggest that a slower *P. caudatum* is easier to strike with *D. nasutum*'s proboscis or *H. vermiculare*'s extruding sting (Baumberg & Hausmann, 2007; Wessenberg & Antipa, 1970). The opposite reaction, an increase in speed, may therefore be maladaptive in the case of the active but random swimmer *D. nasutum* (i.e., increasing the probability of encounter). It is, however, surprising not to observe a speed increase in the presence of *H. vermiculare*, as it would be advantageous to swim faster in front of the sit‐and‐wait predators to decrease the likelihood of being hit. It is more difficult to identify ecological explanations for the initial difference in the speed trends between the two temperatures. Potentially, the speed change could be due to the individuals acclimating to a totally new spatial environment, even if the physiochemical conditions were the same as their stock environment.

In the morphological data, we observed a very clear pattern induced by the presence of *D. nasutum* in the 15°C treatment, with the length:width ratio increasing as the surviving *P. caudatum* individuals developed a more elongated shape (Figure 3). This morphological response to *D. nasutum* exposure corroborates previous results of elongated *P. caudatum* exposed to a nongape‐limited predator (copepods) and confirms the importance of having a more torpedo‐like shape to increase swimming maneuverability and potentially escape the paralyzing effect of the cysts (Uiterwaal et al., 2020). Fast and random swimming movements of *D. nasutum* can result in several physical contacts and bumps with *P. caudatum* before the predator proboscis strikes successfully (Wessenberg & Antipa, 1970); thus, for *P. caudatum*, being able to maneuver away from unsuccessful hits thanks to an elongated shape is advantageous. Surprisingly, this pattern was not observed in the *H. vermiculare* treatment, presumably due to the species distinct movement ecology and vertical distribution. Indeed, *H. vermiculare* individuals mostly crawl and dwell at the bottom of substrates, swimming intermittently but always slowly in the water column (Baumberg & Hausmann, 2007). The attacks of this sit‐and‐wait predator consist of more precise hits rather than multiple contacts like *D. nasutum*. Being more slender might thus be less advantageous for *P. caudatum* in this context.

While *P. caudatum* exposed to *D. nasutum* showed a clear morphological elongation at 15°C, this was not observed at 25°C, a temperature at which the length:width ratio remained stable throughout the 24 h of the experiment (Figure 3). One explanation for these temperature‐dependent patterns may arise from physiological effects on both the predator and prey species surpassing the adaptative advantage of the prey species elongated body shape. Previous work revealed that higher temperatures can decrease the handling time of predators (Robertson & Hammill, 2021; Vucic‐Pestic et al., 2011) and augment their mobility (Beveridge et al., 2010). Indeed, we observed increasing *D. nasutum* swimming velocities in the 25°C treatment (Appendix S1: Figure S6). Altogether, this suggests that the higher escaping capacities of the elongated torpedo‐shaped *P. caudatum* may no longer be an advantage in the face of *D. nasutum*'s elevated predatory activity levels (e.g., increased attack rates and swimming velocities or decreased prey handling time) at 25°C. Moreover, temperature variation seems to induce a rounder cell shape in *Paramecium* (Uiterwaal et al., 2020), potentially making it more flexible in the gas and nutrient exchange rates that change with temperature. Similarly, the high metabolic rate of the ciliates at 25°C might constrain the plasticity of the cell shape, even in the presence of predators. On the other hand, studies on other *Paramecium* species showed that body shape responses are mostly driven by competition and resource levels, while the responses to temperature predominantly concern body size (Tan et al., 2021). Nevertheless, at 25°C, the elongation morphological response of *P. caudatum* could lose its adaptative meaning, and could have been counter selected in the trade‐off between temperature‐ and predator‐optimized cell shapes. While this hypothesis remains speculative, other studies on similar species have also revealed that phenotypic plastic responses can be suppressed or reversed by temperature changes (Luhring et al., 2019; Uiterwaal et al., 2020). Furthermore, in more complex aquatic organisms (e.g., cladocerans), higher temperatures can hamper the intensity of defensive morphological traits expression, as high energy consumption due to faster metabolism might result in insufficient energy for the growth of antipredatory features (e.g., horns, [Qin et al., 2021]). Similarly, we can infer that a fast metabolism does not allow the ciliates to invest enough energy for a significant change in cell shape, despite the presence of the predator's cues potentially inducing it.

To conclude, disentangling the effects of the interaction between two stressors of different natures is most often challenging and counterintuitive (Spake et al., 2023) and requires high‐resolution data to elucidate a mechanistic understanding of the driving processes. Here, by monitoring predator–prey relationships in standardized and temperature‐controlled multispecies microcosms at hourly resolution, we provide novel information about the timing at which prey morphological changes in response to predator exposure are exhibited and modulated by environmental conditions such as temperature. Given that behavioral and morphological trait changes would occur prior abundance changes (Cerini et al., 2023), obtaining such high resolution and multidimensional data is becoming more and more necessary to elucidate the combinatorial effects of multiple stressors exposure, which would be masked at lower temporal resolutions and on single measurements.

## AUTHOR CONTRIBUTIONS

**Francesco Cerini:** Data curation (equal); methodology (supporting); writing – original draft (lead); writing – review and editing (lead). **Duncan O'Brien:** Data curation (equal); formal analysis (lead); writing – review and editing (equal). **Ellie Wolfe:** Conceptualization (lead); data curation (equal); methodology (lead); writing – original draft (equal); writing – review and editing (supporting). **Marc Besson:** Methodology (lead); software (lead); writing – review and editing (equal). **Christopher F. Clements:** Conceptualization (lead); funding acquisition (lead); methodology (equal); project administration (lead); writing – review and editing (supporting).

## CONFLICT OF INTEREST STATEMENT

The authors declare no conflict of interest.

## Supporting information


Appendix S1.

## Data Availability

All code and data are available in the Zenodo repository: https://doi.org/10.5281/zenodo.8246112.
