# Supplementary material for: Phenotypic response to different predator strategies can be mediated by temperature
Source: Ecol Evol. 2023 Aug 31;13(9):e10474. doi: 10.1002/ece3.10474 (PMC10468988; doi:10.1002/ece3.10474)

**APPENDIX 1**

**Manuscript type**: Research Article

**Title:** Phenotypic response to different predator strategies can be mediated by temperature

**Authors:**

Francesco Cerini^1,2^, Duncan O'Brien^2^, Ellie Wolfe^2^, Marc Besson^3^, Christopher F. Clements^2^

*^1^Dipartimento Scienze Ecologiche e Biologiche, Università della Tuscia, Viterbo, Italy*

*^2^School of Biological Sciences, University of Bristol, Bristol, UK*

*^3^Sorbonne Université CNRS UMR Biologie des organismes marins, BIOM, Banyuls-sur-Mer, France*

**Corresponding author**: [francesco.cerini@unitus.it](mailto:francesco.cerini@unitus.it)

**Supplementary Tables**

**Table S1**. Estimated coefficients for the Bayesian hierarchical generalised additive model of mean *Paramecium caudatum* speed through time and across predator and temperature treatments. 15°C and Control predator treatment act as the reference level in this model. Treatmentdidinium: 24 hours exposure to *Didinium nasutum.* Treatmenthomalozoon: 24 hours exposure to *Homalozoon vermiculare*. Treatment25: experiment performed at 25°C. Treatment15: experiment performed at 15°C.

| **Parameter** | **Estimate** | **Est.Error** | **Lower 95% CI** | **Upper 95% CI** | **Rhat** | **Bulk_ESS** | **Tail_ESS** |  |
| --- | --- | --- | --- | --- | --- | --- | --- | --- |
| *random* | | | | | | | | |
| sd(Intercept) | 0.032 | 0.01 | 0.019 | 0.057 | 1 | 2221.96 | 1885.03 |  |
| *fixed* | | | | | | | | |
| Intercept | 0.988 | 0.013 | 0.964 | 1.012 | 1 | 2112.56 | 2297.96 |  |
| treatment25 | -0.163 | 0.008 | -0.179 | -0.147 | 1 | 2521.42 | 2293.74 |  |
| predator_treatmentdidinium | -0.023 | 0.011 | -0.045 | -0.002 | 1 | 2597.82 | 2373.69 |  |
| predator_treatmenthomalozoon | -0.152 | 0.008 | -0.169 | -0.136 | 1 | 2521.92 | 2182.9 |  |
| treatment25:predator_treatmentdidinium | 0.036 | 0.014 | 0.01 | 0.062 | 1 | 2512.81 | 2171.33 |  |
| treatment25:predator_treatmenthomalozoon | 0.115 | 0.011 | 0.094 | 0.137 | 1 | 2483.94 | 2177.37 |  |
| stime_point_1 | -0.773 | 0.713 | -2.121 | 0.606 | 1 | 2512.5 | 2236.29 |  |
| stime_point:treatment15_1 | -2.84 | 0.735 | -4.3 | -1.372 | 1 | 2074.27 | 2459.12 |  |
| stime_point:treatment25_1 | 2.119 | 0.72 | 0.758 | 3.579 | 1 | 2302.37 | 2242.49 |  |
| stime_point:predator_treatmentprey_1 | -0.249 | 0.713 | -1.618 | 1.083 | 1 | 2325.09 | 2387.54 |  |
| stime_point:predator_treatmentdidinium_1 | -0.147 | 0.72 | -1.574 | 1.229 | 1 | 2646.13 | 2245.87 |  |
| stime_point:predator_treatmenthomalozoon_1 | -0.36 | 0.699 | -1.715 | 0.978 | 1 | 2370.23 | 2208.09 |  |
| stime_point:treat_inter15.prey_1 | -1.129 | 0.747 | -2.675 | 0.327 | 1 | 2488.85 | 2253.99 |  |
| stime_point:treat_inter25.prey_1 | 0.892 | 0.759 | -0.572 | 2.389 | 1 | 2452.21 | 1951.36 |  |
| stime_point:treat_inter15.didinium_1 | -0.732 | 0.738 | -2.141 | 0.723 | 1 | 2485.73 | 2293.88 |  |
| stime_point:treat_inter25.didinium_1 | 0.564 | 0.736 | -0.861 | 2.021 | 1 | 2513.09 | 2279.8 |  |
| stime_point:treat_inter15.homalozoon_1 | -0.947 | 0.739 | -2.4 | 0.465 | 1 | 2314.73 | 2245.19 |  |
| stime_point:treat_inter25.homalozoon_1 | 0.595 | 0.732 | -0.839 | 2.067 | 1 | 2608.33 | 2368.92 |  |
| *splines* | | | | | | | | |
| sds(stime_point_1) | 0.307 | 0.249 | 0.011 | 0.94 | 1 | 2179.9 | 2270.93 |  |
| sds(stime_pointtreatment15_1) | 1.706 | 0.557 | 0.914 | 3.071 | 1 | 2246.79 | 2415.38 |  |
| sds(stime_pointtreatment25_1) | 0.5 | 0.292 | 0.031 | 1.205 | 1 | 1786.15 | 1900.98 |  |
| sds(stime_pointpredator_treatmentprey_1) | 0.178 | 0.179 | 0.006 | 0.671 | 1 | 2371.58 | 2361.7 |  |
| sds(stime_pointpredator_treatmentdidinium_1) | 0.256 | 0.225 | 0.009 | 0.811 | 1 | 2228.02 | 2353.27 |  |
| sds(stime_pointpredator_treatmenthomalozoon_1) | 0.168 | 0.159 | 0.006 | 0.568 | 1 | 2316.9 | 2216.75 |  |
| sds(stime_pointtreat_inter15.prey_1) | 0.336 | 0.247 | 0.016 | 0.988 | 1 | 2239.92 | 2231.99 |  |
| sds(stime_pointtreat_inter25.prey_1) | 0.287 | 0.249 | 0.01 | 0.907 | 1 | 2009.31 | 2291.13 |  |
| sds(stime_pointtreat_inter15.didinium_1) | 0.303 | 0.279 | 0.009 | 1.007 | 1 | 2082.95 | 2332.84 |  |
| sds(stime_pointtreat_inter25.didinium_1) | 0.411 | 0.321 | 0.019 | 1.209 | 1 | 2280.09 | 2500.88 |  |
| sds(stime_pointtreat_inter15.homalozoon_1) | 0.594 | 0.413 | 0.026 | 1.541 | 1 | 1935.83 | 2051.45 |  |
| sds(stime_pointtreat_inter25.homalozoon_1) | 0.196 | 0.19 | 0.006 | 0.688 | 1 | 2261.24 | 2308.81 |  |

**Table S2**. Estimated coefficients for the Bayesian hierarchical linear model of *Paramecium caudatum* mean values of width against length, through time and across predator and temperature treatments. 15°C and Control predator treatment act as the reference level in this model. Treatmentdidinium: 24 hours exposure to *Didinium nasutum.* Treatmenthomalozoon: 24 hours exposure to *Homalozoon vermiculare*. Treatment25: experiment performed at 25°. Treatment15: experiment performed at 15°

| **Parameter** | **Estimate** | **Est.Error** | **Lower 95% CI** | **Upper 95% CI** | **Rhat** | **Bulk_ESS** | **Tail_ESS** |
| --- | --- | --- | --- | --- | --- | --- | --- |
| *random* | | | | | | | |
| sd(Intercept) | 0.786 | 0.238 | 0.448 | 1.354 | 1 | 5158.16 | 6239.32 |
| *fixed* | | | | | | | |
| Intercept | 49.306 | 1.135 | 47.053 | 51.541 | 1 | 6307.43 | 6247.92 |
| mean_length_um | 0.147 | 0.005 | 0.138 | 0.156 | 1 | 6137.88 | 6111.5 |
| time_point | 0.126 | 0.121 | -0.111 | 0.36 | 1 | 6434.21 | 6181.56 |
| treatment25 | 0.01 | 0.87 | -1.712 | 1.718 | 1 | 6437.69 | 6144.08 |
| predator_treatmentdidinium | 2.001 | 0.884 | 0.266 | 3.74 | 1 | 6397.62 | 6371.67 |
| predator_treatmenthomalozoon | -0.644 | 0.893 | -2.397 | 1.099 | 1 | 6557.03 | 6354.43 |
| mean_length_um:time_point | 0.002 | 0 | 0.001 | 0.003 | 1 | 6326.23 | 6235.14 |
| mean_length_um:treatment25 | 0.029 | 0.004 | 0.02 | 0.037 | 1 | 6247.61 | 5913.39 |
| time_point:treatment25 | -0.933 | 0.137 | -1.201 | -0.663 | 1 | 6337.43 | 6175.96 |
| mean_length_um:predator_treatmentdidinium | -0.035 | 0.005 | -0.044 | -0.026 | 1 | 6456.44 | 5783.35 |
| mean_length_um:predator_treatmenthomalozoon | -0.013 | 0.004 | -0.021 | -0.004 | 1 | 6394.15 | 6247.13 |
| time_point:predator_treatmentdidinium | 2.35 | 0.188 | 1.987 | 2.729 | 1 | 6180.03 | 6085.04 |
| time_point:predator_treatmenthomalozoon | -0.245 | 0.171 | -0.578 | 0.102 | 1 | 6067.46 | 5973.45 |
| treatment25:predator_treatmentdidinium | -1.638 | 0.904 | -3.374 | 0.122 | 1 | 6292.47 | 5816.41 |
| treatment25:predator_treatmenthomalozoon | 1.196 | 0.908 | -0.558 | 2.973 | 1 | 6309.25 | 6230.88 |
| mean_length_um:time_point:treatment25 | 0.002 | 0.001 | 0.001 | 0.004 | 1 | 6359.55 | 6198.21 |
| mean_length_um:time_point:predator_treatmentdidinium | -0.01 | 0.001 | -0.011 | -0.008 | 1 | 6069.28 | 6177.46 |
| mean_length_um:time_point:predator_treatmenthomalozoon | 0.002 | 0.001 | 0 | 0.003 | 1 | 6266.63 | 6157.36 |
| mean_length_um:treatment25:predator_treatmentdidinium | 0.047 | 0.006 | 0.035 | 0.059 | 1 | 6234.01 | 6247.11 |
| mean_length_um:treatment25:predator_treatmenthomalozoon | 0.027 | 0.005 | 0.016 | 0.037 | 1 | 6332.23 | 6188.22 |
| time_point:treatment25:predator_treatmentdidinium | -1.91 | 0.208 | -2.318 | -1.501 | 1 | 6133.97 | 6133.32 |
| time_point:treatment25:predator_treatmenthomalozoon | 0.485 | 0.197 | 0.099 | 0.864 | 1 | 5935.1 | 6266.65 |
| mean_length_um:time_point:treatment25:predator_treatmentdidinium | 0.007 | 0.001 | 0.005 | 0.009 | 1 | 6063.9 | 6415 |
| mean_length_um:time_point:treatment25:predator_treatmenthomalozoon | -0.003 | 0.001 | -0.005 | -0.001 | 1 | 6274.02 | 6290.36 |

**Supplementary Figures**

**Figure S1.** Population dynamics of the experiments. Counts of tracked individuals in the videos per each replicate (faint lines) and average trend (stronger lines) across the temperature and predators’ treatments. Blue lines are for the experiments performed at 15°C. Red lines for experiments performed at 25°C. *D. nasutum*: 24 hours exposure to *Didinium nasutum. H. vermiculare*: 24 hours exposure to *Homalozoon vermiculare.* Control: 24 hours with prey left alone.

**
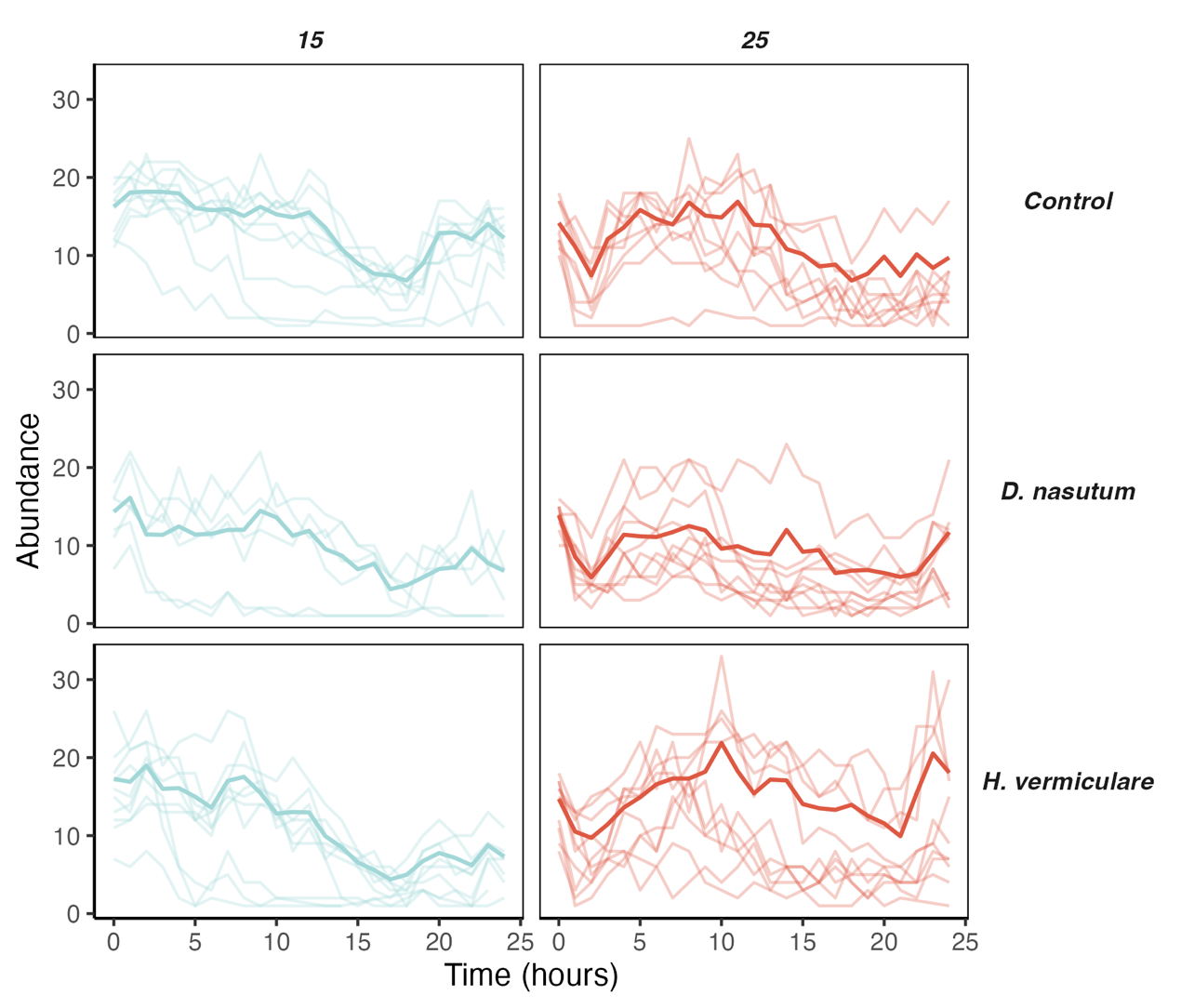
**

**Figure S2.** Trace plots for each parameter of the multilevel generalised additive Bayesian model fitted between the mean swimming speed and the predator and temperature treatments through time. Treatmentdidinium: 24 hours exposure to *Didinium nasutum.* Treatmenthomalozoon: 24 hours exposure to *Homalozoon vermiculare*. Treatment25: experiment performed at 25°. Treatment15: experiment performed at 15°. Visually, a converged fit is indicated by unimodal density plots (left) and ‘well-mixed’/highly overlapping chains (1 to 4, right).


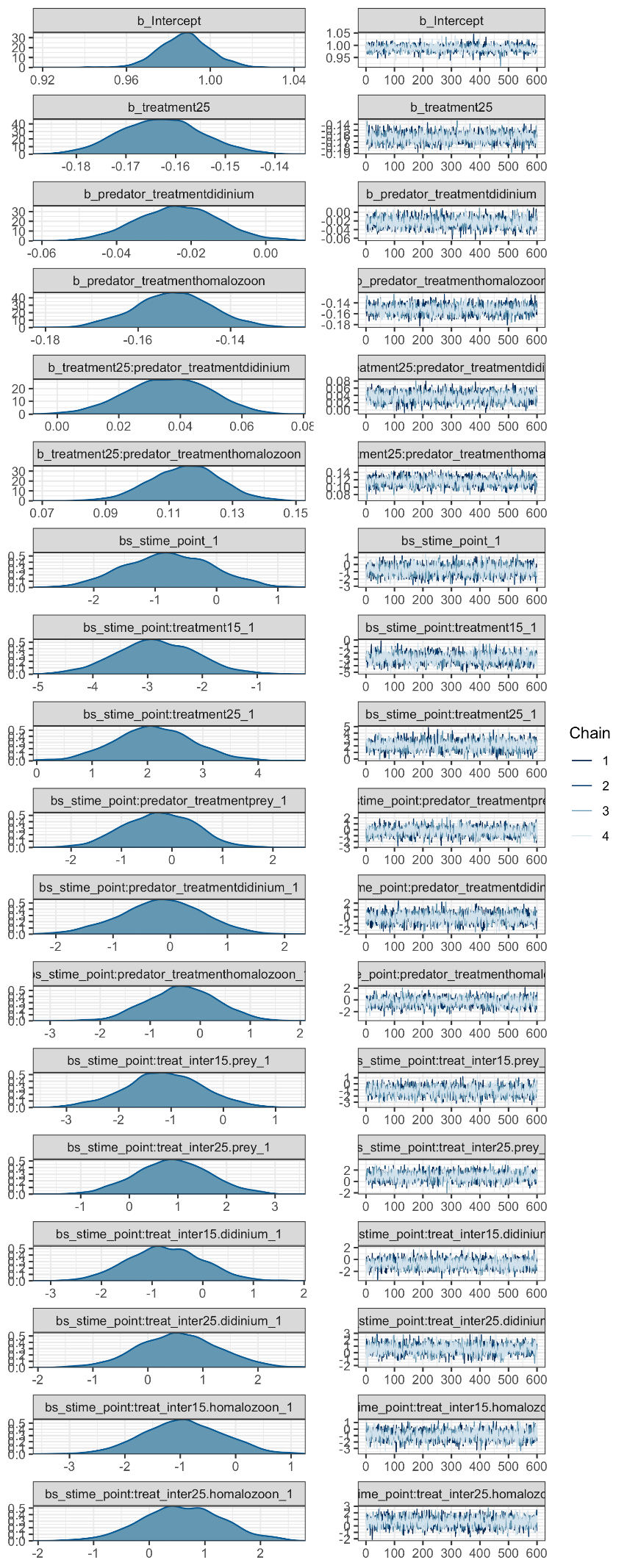


**Figure S1.** **cont**


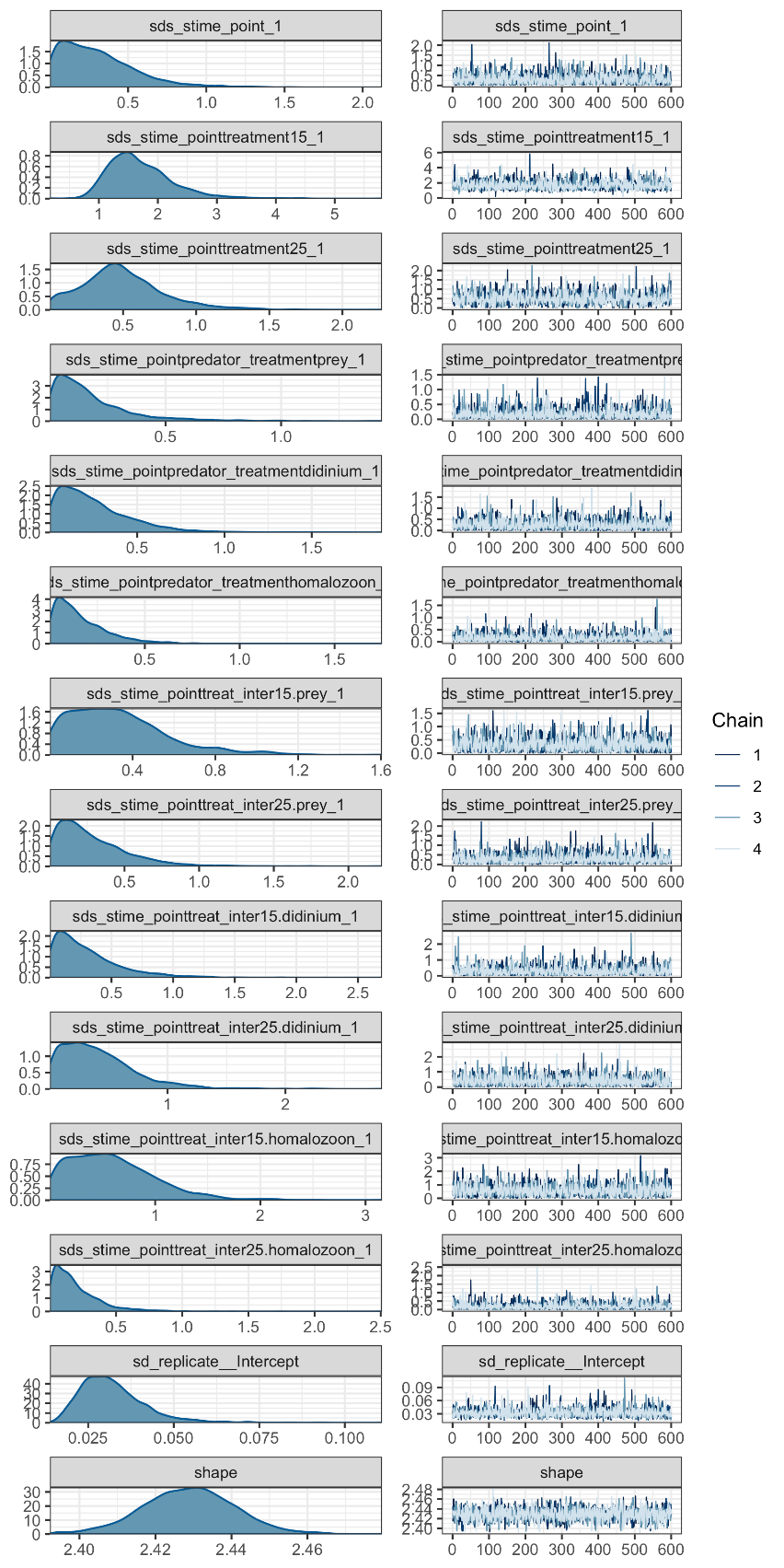


**Figure S3** Trace plots for each parameter of the Bayesian linear mixed effect model fitted between the mean width of the individuals and the mean length, conditional to the predator and temperature treatments through time. Treatmentdidinium: 24 hours exposure to *Didinium nasutum.* Treatmenthomalozoon: 24 hours exposure to *Homalozoon vermiculare*. Treatment25: experiment performed at 25°. Treatment15: experiment performed at 15°. Visually, a converged fit is indicated by unimodal density plots (left) and ‘well-mixed’/highly overlapping chains (1 to 4, right).


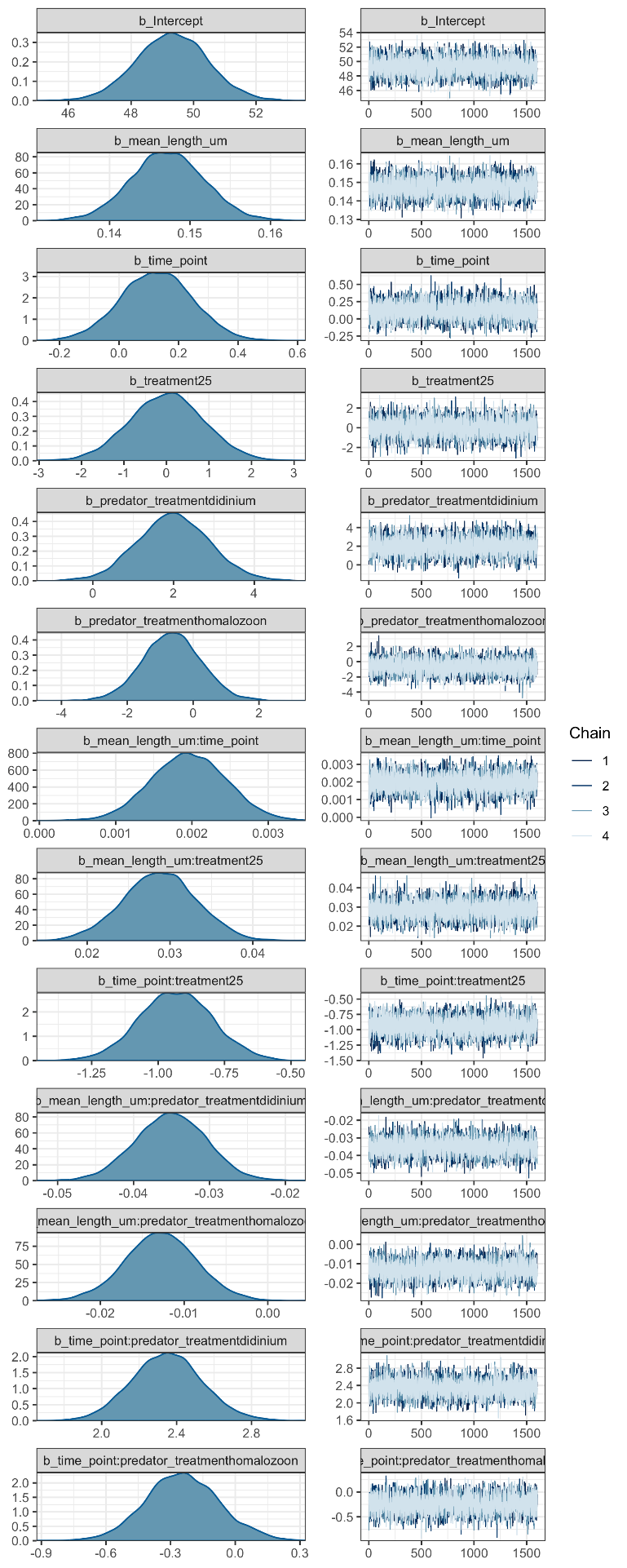


**Figure S2 cont.**


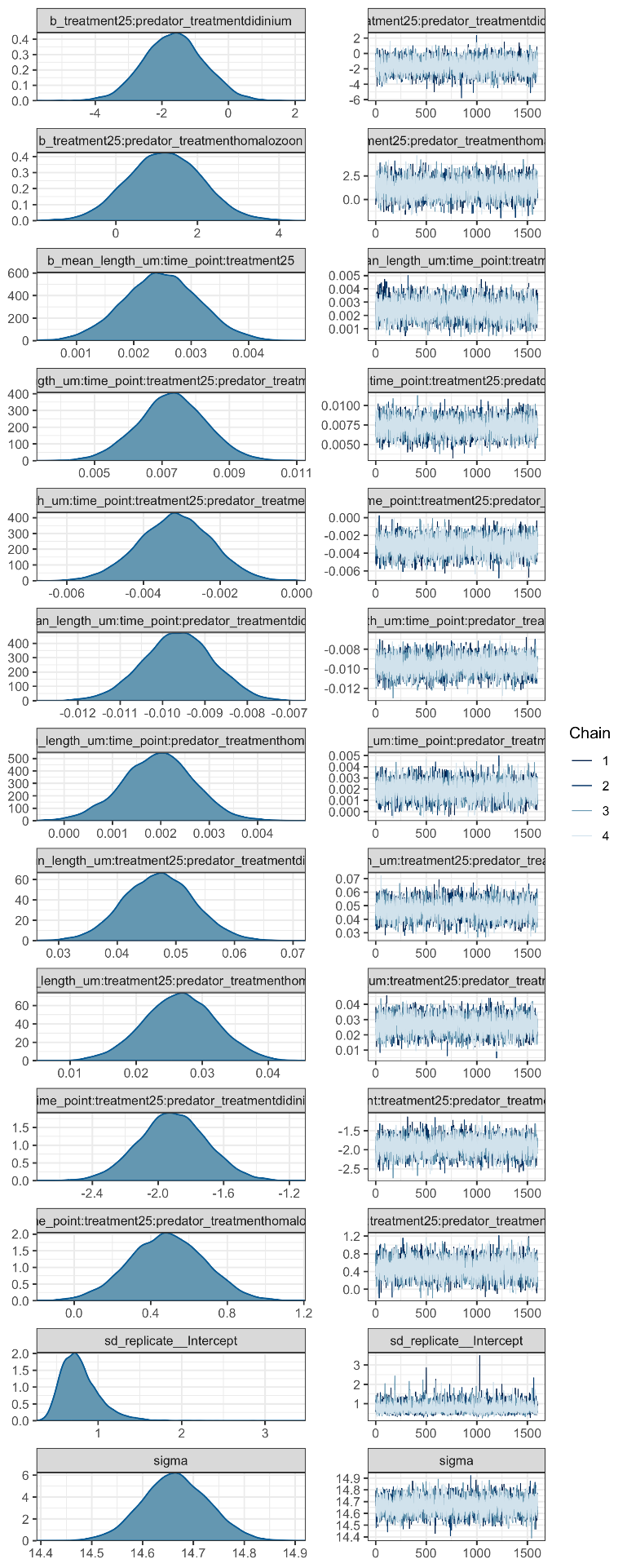


**Figure S4.** Posterior predictive checks of the multilevel generalised additive Bayesian model fitted between the mean swimming speed and the predator and temperature treatments through time. An appropriate fit occurs when y_rep_ reasonably reflects y.


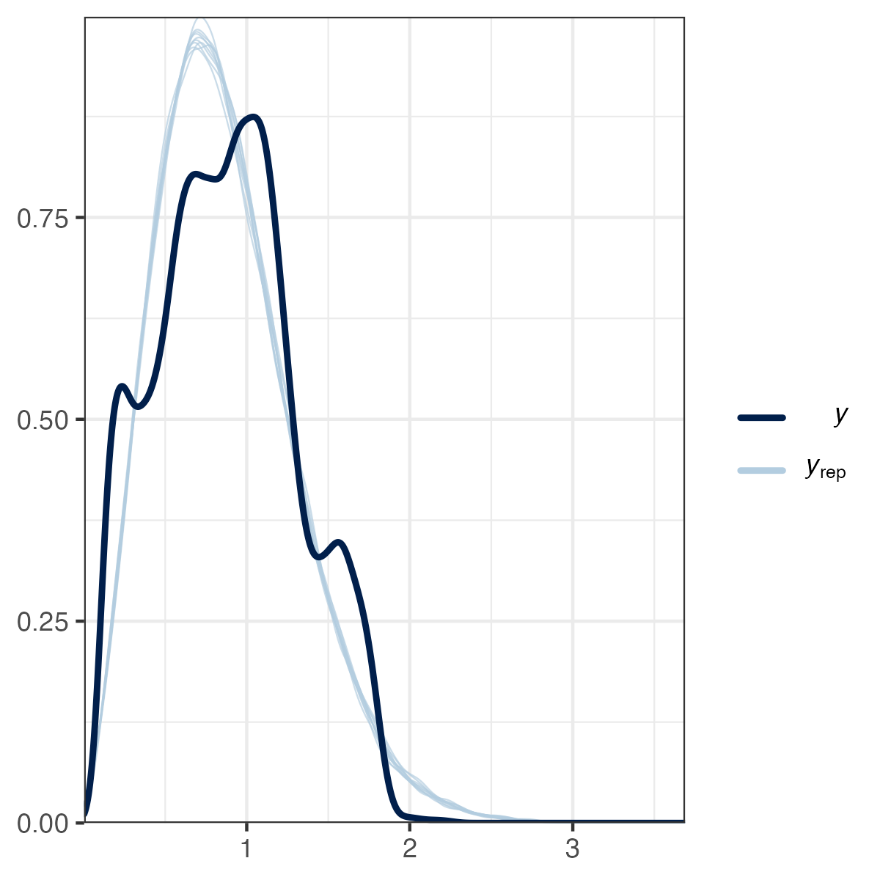


**Figure S5.** Posterior predictive checks of the Bayesian linear mixed effect model fitted between the mean width of the individuals and the mean length, conditional to the predator and temperature treatments through time. An appropriate fit occurs when y_rep_ reasonably reflects y.


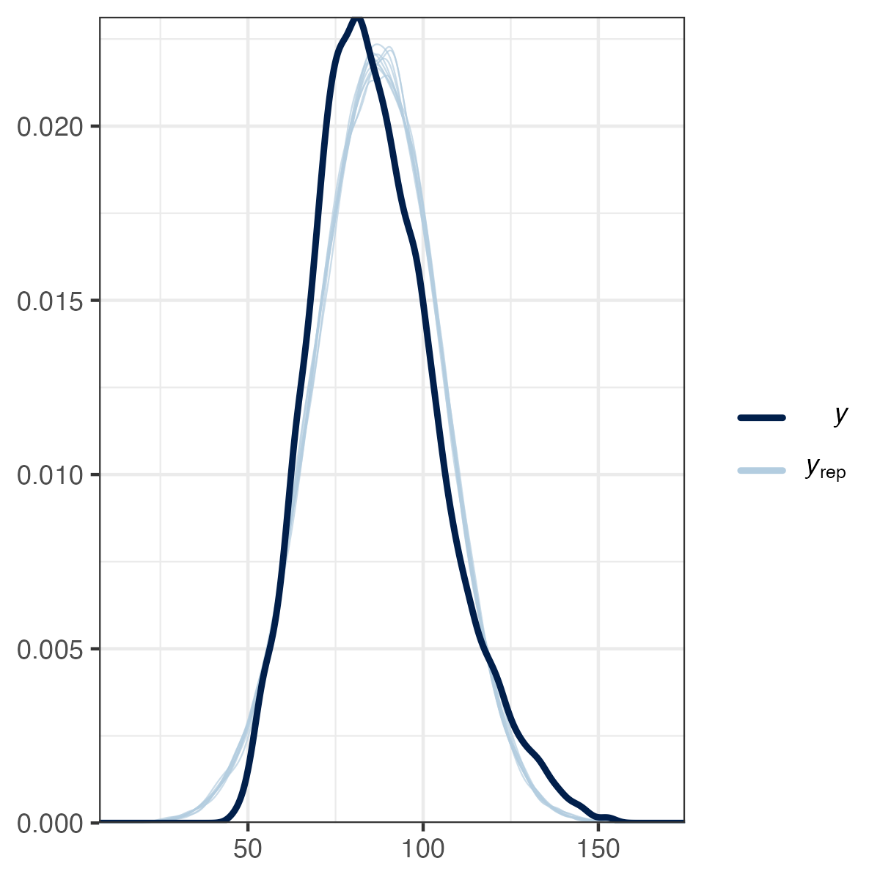


**Figure S6**. Boxplot comparing average movement speeds of the predators in the two temperature treatment, with Wilcoxon test scores to highlight significant difference.


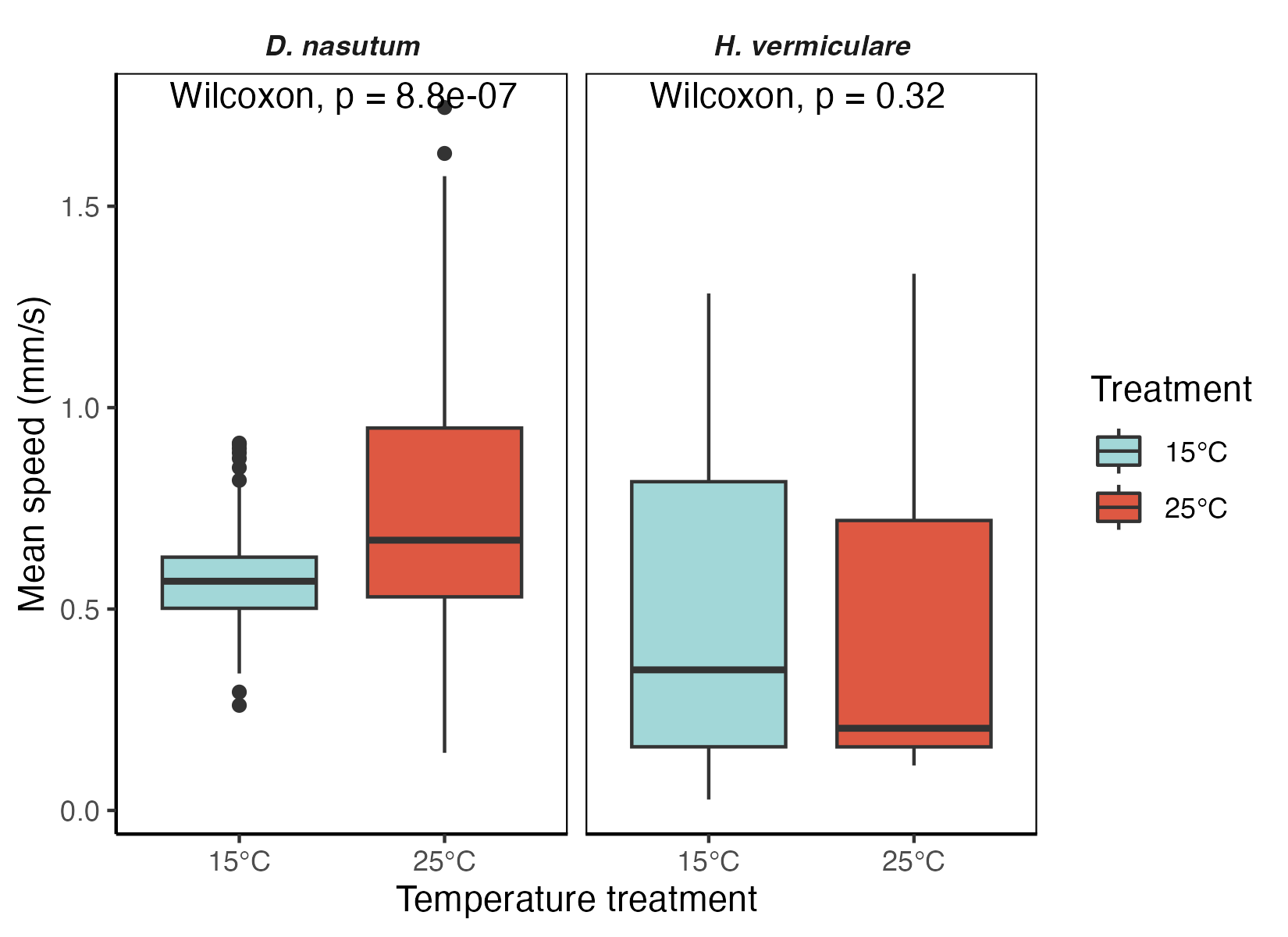

Supplement: Supplementary file 1 — Appendix S1. [file ECE3-13-e10474-s001.docx]
